# Consumption replaces charity: Altruistic consumption behaviors and motivations targeting vulnerable groups—Research based on poverty alleviation consumption in China

**DOI:** 10.3389/fpsyg.2022.933701

**Published:** 2022-08-16

**Authors:** Huiyu Xin, Chenzhuoer Li, Wei Li, Hong Wang, Ping Liu, Shouwei Li

**Affiliations:** ^1^College of Social Sciences, University of Glasgow, Glasgow, United Kingdom; ^2^School of Informatics, The University of Edinburgh, Edinburgh, United Kingdom; ^3^Business School, Sichuan University, Chengdu, China; ^4^Business School, Chengdu University of Technology, Chengdu, China

**Keywords:** charity, vulnerable group, altruistic consumption, altruistic consumption motivation, altruistic marketing strategy, benefit morality motivation

## Abstract

Poverty alleviation consumption, which we call altruistic consumption, has become a new effective way to help vulnerable groups, but there are a few empirical researches on poverty alleviation through consumption. This article takes China's poverty alleviation actions as the research object, investigates and studies the relationship between altruistic consumption motivations and altruistic consumption behaviors that aim for vulnerable groups. It is found that altruistic consumption behavior is mainly affected by benefit group motivation, benefit morality motivation, benefit demander motivation, and benefit supplier motivation. There is a correspondence between the four altruistic consumption motivations and the four altruistic consumption behaviors. The strength of altruistic consumption motivations changes with changes in altruistic buying behavior. The strength of benefit group motivation decreases with the increase in the times of altruistic purchases, while the strength of benefit morality motivation, benefit demander motivation, and benefit supplier motivation increase with the increase in the times of altruistic purchases. Among the four kinds of altruistic consumption motivations that affect the times of altruistic purchases, the benefit demander motivation has a relatively greater influence. The results of this study have important guiding significance for vulnerable groups to formulating targeted proactive marketing strategies, preventing the altruistic consumption relationship dissolution, and realizing sustainable altruistic consumption.

## Introduction

The poverty problem of vulnerable groups is a social issue that is generally concerned by the international community today. The international community is trying to help vulnerable groups get rid of poverty through various channels and methods. Since 2018, a large number of e-commerce platforms such as JD.com, Taobao, and Pinduoduo in China, client apps of a large number of communication, aviation, banking, and insurance enterprises such as China Mobile, Air China, and Bank of China, as well as “We Media” platforms such as Tiktok and Kwai and social platforms such as WeChat and microblog, have successively launched windows to display products from poverty-stricken areas. By encouraging consumers to buy products from such regions, we can help vulnerable groups overcome poverty. In 2020 alone, poverty alleviation products sold through various platforms reached 306.94 billion yuan (about US$46.8 billion), successfully helping a large number of vulnerable groups.

China's model of setting up special sales channels for poverty alleviation products through Internet platforms and encouraging consumers to buy products from poverty-stricken areas, so as to help vulnerable groups overcome poverty, has provided a new way for the international community to eliminate poverty. It is a two-way transaction based on altruism and a market-oriented way of helping others. According to Prosocial Behavior Theory (Batson and Powell, 2003), the behavior of helping others through consumption should be categorized as altruistic behavior; it is a mutually beneficial altruistic behavior that can result in a win–win situation (Sloan, 2015). We call this type of consumption, which takes altruism as its purpose and helps vulnerable groups escape poverty by purchasing the products provided by the vulnerable groups, as altruistic consumption. The core idea of altruistic consumption is to introduce vulnerable groups into the social reproduction system and eliminate poverty through the social-economic cycle of production, distribution, circulation, and consumption and by selling their products through marketization. This is a sustainable way for vulnerable groups to eliminate poverty completely.

The poverty alleviation model launched by China's Internet platform differs from charitable behavior. As a humanitarian act with a long history, although charitable behavior has played an important role in alleviating the poverty of vulnerable groups, there are problems with using charitable methods to solve the poverty problem of the following vulnerable groups: Charity can only temporarily alleviate the poverty situation of vulnerable groups, while cannot completely solve their poverty problem (Eagles, 1993). At the same time, the charity might eventually lead recipients to become heavily dependent on charitable donations or services, turning into toxic charity, and destroying the ecology and vitality of local economic and social development (Lupton, 2011). Market-oriented charitable acts such as marketized philanthropy, as a special charity are considered to have brought a negative impact on the purest core “kindness” of charity (Nickel and Eikenberry, 2009).

In addition to charitable behavior (Thomas and Mcgarty, 2018), most of the research on poverty alleviation focuses on the tourism (Tchouamou and Neelu, 2018; Truong, 2018; Zhao and Xia, 2020), agricultural productivity (Madi et al., 2020), information and communication technology (Galperin and Viecens, 2017; Mbuyisa and Leonard, 2017; Lechman and Popowska, 2022), subsidy policy (Pan et al., 2021), insurance (Gabrah et al., 2020), and education (Brown and James, 2020). These studies mainly focus on improving the efficiency and output of vulnerable groups from the perspective of supply. However, from the perspective of consumption, there is a lack of research on the motivates and behavior of consumer consuming poverty alleviation products. What motivates consumers to engage in altruistic consumption? What impact do these motives have on consumers' altruistic consumption behavior? Is there an evolutionary trend in altruistic consumption motivation in altruistic consumption behavior?

Studying these issues has important theoretical and practical significance for advocating social altruistic consumption behavior, establishing altruistic consumption relationships, and preventing the dissolution of altruistic consumption relationships. It is also a new attempt at expanding prosocial behavior theory to the field of consumption.

## Literature review

The theories related to this article mainly involve charitable behaviors, prosocial behaviors, altruistic behaviors, as well as the times of consumption and recommendation consumption behaviors.

### Charitable behaviors

The meaning of Charity in Old English is “Christian love of one's fellows” (Falus, 2020). Later, the meaning of charity changed to “providing for those in need; generosity and giving” (Merriam-Webster, 2021). Starting from c.1300, charity means “alms, that which is bestowed gratuitously on a person or persons in need” (Online Etymology Dictionary, 2022). This indicates that charitable acts do not need to seek rewards. Charitable acts include providing tangible donations and intangible care. Charitable giving is the act of giving money (Chan and Septianto, 2022), goods (Diederich et al., 2021), or time (Costello and Malkoc, 2022) to the unfortunate, either directly or through a charitable trust or other worthy cause. Charitable care includes such things as visiting imprisoned or homeless people, redeeming captives, educating orphans, and participating in social movements. The most important feature of charitable behavior is that it does not seek any returns.

In the category of charitable consumption behavior. Cause-related marketing is the creation of a mutually beneficial relationship (Chang and Chu, 2020). Its characteristic is that when customers participate in product exchanges that meet organizational and personal goals, the company provides a certain number of charitable donations to specific charities. Using cause-related marketing appropriately not only embodies the corporate social responsibility, but also meets the needs of shareholders to increase profits and market share (Gao et al., 2020). However, its essence is the same as traditional marketing, which is to enhance the corporate image and obtain more benefits.

### Prosocial behaviors

Prosocial behavior is a positive social behavior that intends to help others, and needs to meet two conditions: the behavior is the subject's spontaneous behavior (Shi et al., 2020), including sharing, helping, caring, and comforting (Laguna et al., 2022). The manifestations and scenarios of these behaviors are different, but the essence is basically the same, that is focus on benefiting others (Miles et al., 2021).

In the research on why people have prosocial behaviors, the famous American psychologist Batson et al. (2011) wrote, “*we wish to consider four possible forms of prosocial motivation, each of which can lead a person to benefit-help-someone in need. Each form is defined by its distinct ultimate goal: self-benefit (egoism), benefit another individual (altruism), benefit a group (collectivism), and uphold a moral principle (principlism)*.” Prosocial behavior does not exclude egoism, and it is also believed that both egoists and altruists will adopt prosocial behavior. For example, for the sake of their reputation, egoists take more prosocial behavior in public conditions than in private settings (Rotella et al., 2021). However, in the absence of benefits, egoists are less prosocial than altruists (Rotella, 2020). Regarding the collectivism motivation, Batson (1994) believes that if people value the welfare of a group, and this welfare is threatened or can be enhanced in some way, then they will have a collectivism motivation to promote action to benefit the group. Regarding the principlism motivation, he believes that its ultimate goal is to adhere to a moral principle in order to eliminate the variability of resorting to altruism due to sympathy, while maintaining a consistent moral principle.

This shows that, unlike charitable behaviors, prosocial behaviors include both voluntary helping others and not expecting any rewards, as well as attempts to help others for certain purposes or rewards.

### Altruistic behavior

The concept of altruism has a long history in the fields of philosophy and ethics, and researchers in various fields have tried to explain altruistic behavior from different aspects. The research on altruistic behavior mainly focuses on anthropology, biology, psychology, and sociology. Some researchers believe that the theory of “kin selection” is derived from animal altruism. From an evolutionary biology point of view, Hamilton's Rule (Okasha, 2020) suggests that the genes of altruism can be continued through natural selection. The overall effect may be to increase the number of copies of altruistic behavior genes owned by the next generation, thereby increasing the incidence of altruistic behavior itself. Batson and Powell (2003) studied altruistic behavior from the perspective of motivation, and believed that pure altruistic behavior is based on caring for the interests of others, and does not expect to benefit from helping others. From the perspective of human and society, distinguished it from animal altruism, some scholars have explored theories such as “reciprocal altruism” (Eamonn et al., 2021) and “reputation-seeking” (Rotella et al., 2021). These studies believe that common altruistic behaviors also include reciprocal altruistic behaviors that expect to get rewards after helping others.

In terms of international trade cooperation, Fair Trade is regarded as an act of international altruism. According to the official definition of the World Fair Trade Organization, “*Fair Trade is a trading partnership, based on dialogue, transparency, and respect, that seeks greater equity in international trade. It contributes to sustainable development by offering better trading conditions to, and securing the rights of, marginalized producers and workers*.” In the Fair Trade movement, developed countries in the member state provide better trading conditions and guarantee of the rights of smallholders and workers—especially in developing countries (Re et al., 2020).

### Altruistic behavior on internet

Altruistic behavior also occurs on Internet (Zheng et al., 2021). Altruistic behavior could be seen as an inevitable result of Internet evolution, since the Internet was initiated by volunteers who thought information should be free and easy to access quickly. Since the naissance of the Internet, it has sought a way in which almost infinite informative resources may be integrated and shared (Amichai Hamburger, 2008). Compared with face-to-face communication, people are more willing to help others and more responsible online, for reasons such as anonymity of the Internet and minimization of the authority (Suler, 2004).

### Times of consumption and consumption recommendation behavior

The behavior that a consumer adopts a new idea or new product for the first time is called the attempt purchase behavior or initial purchase behavior. Initial purchase was found to be significant predictor of service retention (Darley and Luethge, 2019). After purchasing a product for the first time, some people will give up continuing to consume, behave as the one-time consumption behavior, and become one-time consumers. While the other part of people will continue to buy the product, which is called repeatedly purchase behavior. For the important factor that affects repeated purchases in the context of online business, Trivedi and Yadav (2020) believe that it is the trust and E-satisfaction. If the experience is satisfactory, the consumer will make a second purchase. If the consumer does not continue to consume after the second purchase, it is called a two-times consumer. If consumers continue to consume three or more times, according to Dimitrieska and Efremova (2021), it is called loyal consumption, or continuous consumption. The core factor affecting continuous consumption is brand (Khamtanet and Jitkuekul, 2021). Some consumers not only consume by themselves, but also recommend others to consume. This behavior is generally called word-of-mouth spread in academics (see, e.g., Bastos and Moore, 2021). The core factor affecting word-of-mouth spread is consumers' satisfaction and their relationship with the recommendation (Leon and Choi, 2020; Oraedu, 2020). The purpose of studying the times of consumption is to find motivations for one-time consumption, two-times consumption, continuous consumption, and recommendation consumption, so as to realize the transformation from one-time consumption to continuous consumption and loyal consumption, to encourage consumers to recommend others to consume, prevent the dissolution of altruistic consumption relationship, and realize the sustainability of altruistic consumption.

## Research framework and hypothesis

### Research structure model

Vulnerable groups have low income, endure poverty, and are psychologically vulnerable (Shu, 2016). Some researchers believe that vulnerable groups refer to social groups that cannot maintain basic living standards for themselves and for their family by relying on their strengths or abilities and thus need external support (Zheng and Li, 2003). The *vulnerable groups* referred to in this article refer to countries, regions, ethnic minorities, low-income groups that have the ability to yield but are in a state of poverty and backwardness, and groups that are economically damaged due to disasters. We call the products provided by vulnerable groups and consumed by altruistic consumers as *altruistic consumption products*, and marketing for altruistic consumption products as *altruistic marketing*.

According to prosocial behavior theory the motivation of prosocial behavior includes collectivism, egoism, altruism, and principlism (Batson et al., 2011). The altruistic consumption behavior studied in this study is also a kind of prosocial behavior, it should also include these four basic motivations. In Batson's theory, collectivism motivation refers to the motivation to benefit a specific group, including the group which the implementer of prosocial behavior belongs. In our previous research, we found that most of the altruistic consumption behaviors are triggered based on the initiative of the group to which the consumer belongs or some specific groups. In addition to helping vulnerable groups, consumers' motivation also lies in maintaining the group's reputation and values. In this article, we call the consumption motivation that benefits to groups in the process of altruistic consumption as the *benefit group altruistic consumption motivation*, abbreviate it as *benefit group motivation*. In Batson's theory, egoism refers to self-benefit, or benefit the implementer of prosocial behavior self. In the relationship of altruistic consumption, self-benefit is actually benefits to the consumers who consume altruistic products. In this article, we call the consumption motivation that are benefits to the consumers of altruistic product consumption as the *benefit demander altruistic consumption motivation*, abbreviate it as *benefit demander motivation*. In Batson's theory, altruism refers to benefit another individual. In the relationship of altruistic consumption, “another individual” is actually the supplier (i.e., vulnerable groups) of altruistic products. In this article, we call the consumption motivation that is benefits to the supplier of altruistic products as the *benefit supplier altruistic consumption motivation*, abbreviate it as the *benefit supplier motivation*. In Batson's theory, principlism refers to benefit a moral principle. In the relationship of altruistic consumption, it is mainly manifested as the promotion of a kind of social virtue. In this article, we call the consumption motivation that is benefits to social morality as the *benefit morality altruistic consumption motivation*, abbreviate it as the *benefit morality motivation*. In order to facilitate research, we collectively refer to these four altruistic consumption motivations as 4B altruistic consumption motivations.

According to the literature (Tucker, 1964; Bass, 1969; Blythe, 2006; Zhu, 2014) and our previous research, the altruistic consumption behavior in this article can be divided into one-time altruistic consumption behavior, two-times altruistic consumption behavior, continuous altruistic consumption behavior, and altruistic recommendation consumption behavior. What is the relationship between these four kinds of consumption behaviors and the four kinds of consumption motivations? Do these four kinds of consumption behaviors have different dominant consumption motivations, respectively? Is there an evolutionary trend for the four kinds of altruistic consumption motivations in the four kinds of consumption behaviors?

### Research hypothesis

#### The relationship between altruistic consumption behavior and altruistic consumption motivation

Consumers' motivation for consumption is the internal driving force that motivates consumers to implement consumption behaviors. There is a causal relationship between consumption motivation and consumption behavior. Altruistic consumption motivation should also be the internal driving force of altruistic consumption behavior, and there should also be a causal relationship between them. Consumption behavior not only includes the behavior that consumers purchase by themselves, but also includes the behavior that recommends others to purchase. It can be seen that altruistic consumption behavior should also include altruistic purchase behavior and altruistic recommendation consumption behavior. Therefore, no matter it is the altruistic purchase behavior of consumers themselves or the altruistic recommendation consumption behavior of recommending others to purchase, there should be an influential relationship between the altruistic consumption motivations.

Accordingly, the following hypotheses are proposed:

**H1**_**a**_: There is an influential relationship between the altruistic purchase behavior of the vulnerable groups and the consumer's altruistic consumption motivation
**H1**_**b**_: There is an influential relationship between the altruistic recommendation consumption behavior of the vulnerable groups and the consumer's altruistic consumption motivation

#### The relationship between four kinds of altruistic consumption behaviors and four kinds of altruistic consumption motivations

Whether it is one-time altruistic consumption behavior, two-times altruistic consumption behavior, continuous altruistic consumption behavior, or altruistic recommendation consumption behavior, all are altruistic consumption behaviors, and altruistic consumption behavior is a prosocial behavior. According to the research of Batson et al. (2011), prosocial behavior is affected by four kinds of motivations, so these four kinds of altruistic consumption behaviors should also be affected by four kinds of prosocial behavior motivations. The benefit demander motivation, benefit supplier motivation, benefit morality motivation, and benefit group motivation proposed in this article are based on the four kinds of prosocial behavior motivations proposed by Batson et al. (2011), combined with the characteristics of altruistic consumption, which are the embodiment of the four kinds of prosocial behavior motivations in the field of consumption. In this sense, these four kinds of altruistic consumption behaviors should be affected by four kinds of altruistic consumption motivations.

If consumers' altruistic consumption motivation is the same, their altruistic consumption behaviors should be consistent. However, in the pre-survey phase of our questionnaire, we studied the altruistic consumption behavior of 198 consumers and found that some consumers only purchase one-time, some consumers purchase two-times, some consumers purchase multiple times, and some consumers not only purchase for themselves, but also recommend others for altruistic consumption. It can be seen that there are significant differences in the altruistic consumption behavior of consumers. Since consumers' behaviors are inconsistent, the strengths of the four kinds of altruistic consumption motivations that affect consumers' altruistic consumption behavior should also be different. Each consumer behavior may have different dominant motivations.

Accordingly, the following hypotheses are proposed:

**H2**_**a**_: One-time altruistic consumption behavior is affected by four kinds of altruistic consumption motivations, and there are differences in the strength of the influence, with one or more dominant motivations.
**H2**_**b**_: Two-times altruistic consumption behavior is affected by four kinds of altruistic consumption motivations, and there are differences in the strength of the influence, with one or more dominant motivations.
**H2**_**c**_: Continuous altruistic consumption behaviors are affected by the four kinds of altruistic consumption motivations, and there are differences in the strength of the influence, with one or more dominant motivations.
**H2**_**d**_: Altruistic recommendation consumption behavior is affected by the four kinds of altruistic consumption motivations, and there are differences in the strength of the influence, with one or more dominant motivations.

#### The evolutionary trend of altruistic consumption motivations' influence on altruistic consumption behavior

The essence of altruistic consumption is consumption. When consumers make purchase decisions, they will choose solutions with greater customer perceived value (Zeithaml, 1988). This means that consumers will choose products that better meet their own needs. If consumers do not consider their own needs, it is pure altruistic behavior, rather than reciprocal altruistic consumption behavior. Therefore, among the four kinds of altruistic consumption motivations, the most basic motivation should be the consumer's benefit demander motivation, which means that if altruistic consumers are to continuously purchase altruistic products, they must meet their basic demand for products. Therefore, the benefit demander motivation should be the one that has the greatest influence on consumers' times of altruistic purchases among the four kinds of motivations.

Accordingly, the following hypotheses are proposed:

**H3**_**a**_: Among the four kinds of altruistic consumption motivations that affect the times of altruistic purchases, the benefit demander motivation has relatively greater influence.

Our pre-survey research found that the altruistic consumption behavior of ordinary consumers is mostly influenced by the group, especially by the group's initiative. Therefore, the influence of benefit group motivation in the early stage of altruistic consumption should be relatively large. With the increase in the times of purchases, the focus of consumers' attention has shifted to the value of altruistic products and their consumption behaviors to vulnerable groups and society. Therefore, the strength of benefit group motivation should gradually decrease, while the strength of benefit morality, benefit demander, and profit supplier motivation should gradually increase.

Accordingly, the following hypotheses are proposed:

**H3**_**b**_: The strength of the influence of benefit group motivation decreases with the increase in the times of altruistic purchases, while the strength of the influence of benefit morality motivation, benefit demander motivation, and benefit supplier motivation increases with the increase in the times of altruistic purchases.

## Methodology

### Sample selection and data sources

This study takes the altruistic product consumers participating in poverty alleviation consumption as the survey object, and obtains data through online and offline questionnaire survey. The survey time is from March to May 2021. The content of the questionnaire included the interviewee's basic information about altruistic consumption and the research scale. The variables were measured using the Likert 7 points scale, and the interviewees scored according to their actual consumption.

### Measures

There are eight variables in the scale of this research. There are three items for the variables related to altruistic consumption behavior to measure the times of purchases and altruistic recommendation consumption behavior of interviewees. There are four variables about the motivation of altruistic purchase, and each variable is designed with three items to measure the strength of the altruistic purchase motivation. Regarding the altruistic recommendation consumption motivation, there are the same four variables, and each variable is designed with three items to measure the strength of the altruistic recommendation consumption motivation. The design of the measurement items draws on the scale design by Oyserman (1993), Gudykunst et al. (1996), Leung and Kim (1997), and Curry et al. (2018) [(as cited in Taras et al., 2014)], combined with the characteristics of altruistic products and the background of poverty alleviation we surveyed, and made adjustments and supplements and improvements on the scale. Through three experimental tests, factor analysis, and reliability and validity analysis were carried out, the scale was revised, and the final formal scale of altruistic consumption behavior and motivation in this article was formed.

### Questionnaire pre-survey

After the questionnaire was formed, 198 questionnaires were formally distributed for pre-survey. We use IBM SPSS Statistics 26 to perform KMO and Bartlett's Test on the scale. The results show that the KMO value range of 8 variables is 0.729–0.769, which is higher than the accepted standard of 0.5, and the Bartlett's sphere test's Sig. < 0.001, reaches a significant level, indicating that the sample data is suitable for factor analysis. We perform exploratory factor analysis (EFA), using principal component analysis to extract common factors, and retain items with common factor variance > 0.7. According to the scholar Kaiser (1960) view, select factors with eigenvalues > 1 to determine the number of factors. After performing principal component analysis on 8 variables, each variable has only one eigenvalue > 1, which means there is no transposition situation, and its explained variance reaches 82.568–95.067%, which shows that all 8 variables in this questionnaire are single-dimensional indicators.

Finally, we analyzed the reliability of the scale and found that the reliability coefficient (Cronbach's α value) of the variable was 0.894–0.974, indicating that the measurement item has high reliability and stability. Therefore, it is determined that the design of the questionnaire is reasonable and the questionnaire can be formally distributed. Since the questionnaire was not revised after the test, the 198 questionnaires from the pre-survey were included in the official statistics.

## Results

### Sample characteristics

This study adopts a bricks-and-clicks survey method, using the “Wenjuanxing” online survey platform to distribute and collect questionnaires online, and doing field surveys by distributing paper questionnaires, the survey scope covers most areas of China. Collecting data through two distribution channels at the same time that ensured the sample data is sufficient on the one hand, and guaranteed the diversity and reliability of the sample sources on the other hand. A total of 1,591 questionnaires were collected in the survey, 254 invalid questionnaires with incomplete information, conflicting information, or not in the scope of the survey were excluded, and 1,337 valid questionnaires were obtained. The statistical characteristics of the sample of valid survey objects are shown in Table 1.

**Table 1:** Sample basic information.

	**Feature**	**Frequency**	**%**
Gender	Male	678	50.71
	Female	659	49.29
Age	Under 18	6	0.45
	18–25	169	12.64
	26–30	310	23.19
	31–40	346	25.88
	41–50	282	21.09
	51–60	186	13.91
	60 and above	38	2.84
Educational	High school and below	140	10.47
background	Associate degree	269	20.12
	Bachelor degree	573	42.86
	Postgraduate degree and above	355	26.55
Career	Student	119	8.90
	Private company employees	422	31.56
	Self-employed individual	100	7.48
	Government and public institution employees	415	31.04
	Freelancer	100	7.48
	Retired and honorable discharge personnel	75	5.61
	Others	106	7.93
Average	3,000 RMB and below	192	14.36
monthly	3,001–6,000 RMB	328	24.53
income	6,001–10,000 RMB	459	34.33
	10,000 RMB and above	358	26.78

### Reliability and validity analysis

This article mainly uses exploratory factor analysis (EFA) to test the validity (see Table 2). The KMO values of the variables are all > 0.7, higher than the minimum standard of 0.5, and *p* < 0.001 (significant value) in Bartlett's sphericity test, which indicates that the correlation between the variable measurement items is strong. Principal component analysis (PCA) was performed on 8 variables, and items with common factor variance > 0.7 were retained. Factors with eigenvalues > 1, their explained variance lie between 75.674 and 94.488%. Therefore, it can be considered that the scale has good validity.

**Table 2:** Reliability and validity test.

**Measured variable**	**KMO**	**Cronbach's α**	**Percentage of explained variance**	**Bartlett's sphericity test**
Benefit group consumption motivation	0.751	0.924	86.929	*p* < 0.001 Reject sphericity hypothesis
Benefit morality consumption motivation	0.765	0.939	89.210	*p* < 0.001 Reject sphericity hypothesis
Benefit demander consumption motivation	0.721	0.881	80.973	*p* < 0.001 Reject sphericity hypothesis
Benefit supplier consumption motivation	0.781	0.968	94.001	*p* < 0.001 Reject sphericity hypothesis
Benefit group recommendation consumption motivation	0.767	0.955	91.801	*p* < 0.001 Reject sphericity hypothesis
Benefit morality recommendation consumption motivation	0.768	0.935	88.596	*p* < 0.001 Reject sphericity hypothesis
Benefit demander recommendation consumption motivation	0.702	0.836	75.674	*p* < 0.001 Reject sphericity hypothesis
Benefit supplier recommendation consumption motivation	0.778	0.971	94.488	*p* < 0.001 Reject sphericity hypothesis

In the reliability test conducted in this study (see Table 2), the reliability coefficients (Cronbach's α value) of the eight variables are all > 0.7, therefore the scale can be considered to have good reliability.

### Hypothesis verification

Using Stata SE 15.0 to analyze the data, through the hypothesis verification, we found that the hypothesis has been verified.

#### The relationship between altruistic consumption behavior and altruistic consumption motivation

We have studied the influence of four kinds of altruistic consumption motivations and four kinds of altruistic recommendation consumption motivations on altruistic consumption, and the statistical results are shown in Table 3.

**Table 3:** Distribution of variable data.

**Altruistic consumption motivation**		**Scale anchors**	**Freq**.	**Percent**	**Cum**
Altruistic	Benefit group	<4	301	22.51	22.51
purchase	motivation	4	123	9.20	31.71
motivation		>4	913	68.29	100.00
	Benefit	<4	166	12.42	12.42
	morality	4	64	4.79	17.20
	motivation	>4	1,107	82.79	100.00
	Benefit	<4	134	10.02	10.02
	demander	4	91	6.81	16.83
	motivation	>4	1,112	83.17	100.00
	Benefit	<4	206	15.41	15.41
	supplier	4	69	5.16	20.57
	motivation	>4	1,062	79.43	100.00
Altruistic	Benefit group	<4	252	32.06	32.06
recommendation	motivation	4	92	11.70	43.77
consumption motivation		>4	442	56.24	100.00
	Benefit	<4	66	8.40	8.40
	morality	4	52	6.62	15.01
	motivation	>4	668	84.98	100.00
	Benefit	<4	55	7.00	7.00
	demander	4	33	4.20	11.20
	motivation	>4	698	88.80	100.00
	Benefit	<4	32	4.07	4.07
	supplier	4	40	5.09	9.16
	motivation	>4	714	90.84	100.00

There is an influential relationship between the altruistic purchase behavior for the vulnerable group and the consumers' altruistic consumption motivation. Our investigation is about the motivations of consumers who have purchased altruistic products. The same questionnaire item can only measure the motivation of purchasers, but not the motivation of non-purchasers. Therefore, we cannot perform inferential statistics, but can only perform descriptive statistics.

We calculated the data of the benefit group consumption motivation item in the scale and found that 22.51% of consumers disagree, 9.20% of consumers maintain a neutral attitude, and 68.29% of consumers agree. This shows that when consumers purchase altruistic products, most consumers have obvious benefit group motivation, that is, there is an influential relationship between altruistic purchase behavior and benefit group consumption motivation.

We calculated the data of the benefit morality consumption motivation item in the scale and found that 12.42% of consumers disagree, 4.79% of consumers maintain a neutral attitude, and 82.79% of consumers agree. This shows that when consumers purchase altruistic products, most consumers have obvious benefit morality motivation, that is, there is an influential relationship between altruistic purchase behavior and benefit morality consumption motivation.

We calculated the data of the benefit demander consumption motivation item in the scale and found that 10.02% of consumers disagree, 6.81% of consumers maintain a neutral attitude, and 83.17% of consumers agree. This shows that when consumers purchase altruistic products, most consumers have obvious benefit demander motivation, that is, there is an influential relationship between altruistic purchase behavior and benefit demander consumption motivation.

We calculated the data of the benefit supplier consumption motivation item in the scale and found that 15.41% of consumers disagree, 5.16% of consumers maintain a neutral attitude, and 79.43% of consumers agree. This shows that when consumers purchase altruistic products, most consumers have obvious benefit supplier motivation, that is, there is an influential relationship between altruistic purchase behavior and benefit supplier consumption motivation.

Therefore, we believe that the altruistic purchase behavior for the vulnerable group is related to the consumer's altruistic consumption motivation, that is, hypothesis H1_a_ holds.

There is an influential relationship between the altruistic recommendation consumption behavior for the vulnerable group and the consumers' altruistic consumption motivation. Our investigation is about the motivations of consumers who have purchased altruistic products. The same questionnaire item can only measure the motivation of purchasers, but not the motivation of non-purchasers. Therefore, we cannot perform inferential statistics, but can only perform descriptive statistics.

We calculated the data of the benefit group recommendation consumption motivation item in the scale and found that 32.06% of consumers disagree, 11.70% of consumers maintain a neutral attitude, and 56.24% of consumers agree. This shows that when consumers recommend others to consume altruistic products, most consumers have obvious benefit group motivation, that is, there is an influential relationship between altruistic recommendation consumption behavior and benefit group consumption motivation.

We calculated the data of the benefit morality recommendation consumption motivation item in the scale and found that 8.40% of consumers disagree, 6.62% of consumers maintain a neutral attitude, and 84.98% of consumers agree. This shows that when consumers recommend others to consume altruistic products, most consumers have obvious benefit morality motivation, that is, there is an influential relationship between altruistic recommendation consumption behavior and benefit morality consumption motivation.

We calculated the data of the benefit demander recommendation consumption motivation item in the scale and found that 7.00% of consumers disagree, 4.20% of consumers maintain a neutral attitude, and 88.80% of consumers agree. This shows that when consumers recommend others to consume altruistic products, most consumers have obvious benefit demander motivation, that is, there is an influential relationship between altruistic recommendation consumption behavior and benefit demander consumption motivation.

We calculated the data of the benefit supplier recommendation consumption motivation item in the scale and found that 4.07% of consumers disagree, 5.09% of consumers maintain a neutral attitude, and 90.84% of consumers agree. This shows that when consumers recommend others to consume altruistic products, most consumers have obvious benefit supplier motivation, that is, there is an influential relationship between altruistic recommendation consumption behavior and benefit supplier consumption motivation.

Therefore, we believe that the altruistic recommendation consumption behavior for the vulnerable group is related to the consumer's altruistic consumption motivation, that is, hypothesis H1_b_ holds.

#### The relationship between four kinds of altruistic consumption behaviors and four kinds of altruistic consumption motivations

We studied the relationship between one-time consumption behavior, two-times consumption behavior, continuous consumption behavior, and altruistic recommendation consumption behavior and four kinds of altruistic consumption motivations. The statistical results are shown in Table 4.

**Table 4:** Mean value and *t*-test of variable data.

	**Motivation**	**Behavior**			
		**One-time consumption behavior**	**Two-times consumption behavior**	**Continuous consumption behavior**	**Altruistic recommendation consumption behavior**
Mean value	Benefit group	5.292	4.854	4.714	4.447
	Benefit morality	5.102	5.272	5.941	5.681
	Benefit demander	4.898	5.573	5.659	5.622
	Benefit supplier	5.005	5.301	5.790	5.993
The mean values'	Benefit group	*p* < 0.05		*p* < 0.001	
significance of *t*-test	Benefit morality				
	Benefit group	*p* < 0.001	*p* < 0.001		
	Benefit demander				
	Benefit group	*p* < 0.01			*p* < 0.001
	Benefit supplier				
	Benefit morality		*p* < 0.01	*p* < 0.001	
	Benefit demander				
	Benefit morality			*p* < 0.001	*p* < 0.001
	Benefit supplier				
	Benefit demander		*p* < 0.05		*p* < 0.001
	Benefit supplier				

One-time altruistic consumption behavior is affected by four kinds of altruistic consumption motivations, and there are differences in the strength of the influence, and there is a dominant motivation. Analyzing the mean value of the data about the four kinds of altruistic consumption motivations for consumers who have only purchased altruistic products one time, it is found that the mean value of benefit group consumption motivation is 5.292, the average value of benefit morality consumption motivation is 5.101, the average value of benefit demander consumption motivation is 4.898, and the mean value of benefit supplier consumption motivation is 5.005. The data of these consumers on the four kinds of altruistic consumption motivations are all > 4, indicating that these consumers do have four kinds of altruistic consumption motivations, which means one-time altruistic consumption behavior is related to the four kinds of altruistic consumption motivations. In the mean value analysis, it can be seen that the mean value of the benefit group consumption motivation is the largest. We perform *t*-tests on the benefit group consumption motivation with benefit morality consumption motivation, benefit demander consumption motivation, and benefit supplier consumption motivation, respectively. The results found that the difference in the mean value between benefit group consumption motivation and the other three kinds of altruistic consumption motivations was significant (*p* < 0.05), indicating that benefit group motivation has the greatest influence among the four kinds of altruistic consumption motivations that affect one-time altruistic consumption behavior. Therefore, hypothesis H2_a_ holds.

Two-times altruistic consumption behavior is affected by four kinds of altruistic consumption motivations, and there are differences in the strength of the influence, and there is a dominant motivation. Analyzing the mean value of the data about the four kinds of altruistic consumption motivations for consumers who have only purchased altruistic products two times, it is found that the mean value of benefit group consumption motivation is 4.854, the average value of benefit morality consumption motivation is 5.271, the average value of benefit demander consumption motivation is 5.572, and the mean value of benefit supplier consumption motivation is 5.301. The data of these consumers on the four kinds of altruistic consumption motivations are all > 4, indicating that these consumers do have four kinds of altruistic consumption motivations, which means two-times altruistic consumption behavior is related to the four kinds of altruistic consumption motivations. In the mean value analysis, it can be seen that the mean value of the benefit demander consumption motivation is the largest. We perform *t*-tests on the benefit demander consumption motivation with benefit group consumption motivation, benefit morality consumption motivation, and benefit supplier consumption motivation, respectively. The results found that the difference in the mean value between benefit demander consumption motivation and the other three kinds of altruistic consumption motivations was significant (*p* < 0.05), indicating that benefit demander motivation has the greatest influence among the four kinds of altruistic consumption motivations that affect two-times altruistic consumption behavior. Therefore, hypothesis H2_b_ holds.

Continuous altruistic consumption behavior is affected by four kinds of altruistic consumption motivations, and there are differences in the strength of the influence, and there is a dominant motivation. Analyzing the mean value of the data about the four kinds of altruistic consumption motivations for consumers who have purchased altruistic products continuously, it is found that the mean value of benefit group consumption motivation is 4.713, the average value of benefit morality consumption motivation is 5.941, the average value of benefit demander consumption motivation is 5.659, and the mean value of benefit supplier consumption motivation is 5.790. The data of these consumers on the four kinds of altruistic consumption motivations are all > 4, indicating that these consumers do have four kinds of altruistic consumption motivations, which means continuous altruistic consumption behavior is related to the four kinds of altruistic consumption motivations. In the mean value analysis, it can be seen that the mean value of the benefit morality consumption motivation is the largest. We perform *t*-tests on the benefit morality consumption motivation with benefit group consumption motivation, benefit demander consumption motivation, and benefit supplier consumption motivation, respectively. The results found that the difference in the mean value between benefit morality consumption motivation and the other three kinds of altruistic consumption motivations was significant (*p* < 0.05), indicating that benefit morality motivation has the greatest influence among the four kinds of altruistic consumption motivations that affect continuous altruistic consumption behavior. Therefore, hypothesis H2_c_ holds.

Altruistic recommendation consumption behavior is affected by four kinds of altruistic consumption motivations, and there are differences in the strength of the influence, and there is a dominant motivation. Analyzing the mean value of the data about the four kinds of altruistic consumption motivations for consumers who not only purchased altruistic products themselves but also recommend others to purchase altruistic products, it is found that the mean value of benefit group recommendation consumption motivation is 4.446, the average value of benefit morality recommendation consumption motivation is 5.681, the average value of benefit demander recommendation consumption motivation is 5.622, and the mean value of benefit supplier recommendation consumption motivation is 5.992. The data of these consumers on the four kinds of altruistic recommendation consumption motivations are all > 4, indicating that these consumers do have four kinds of altruistic recommendation consumption motivations, which means altruistic recommendation consumption behavior is related to the four kinds of altruistic recommendation consumption motivations. In the mean value analysis, it can be seen that the mean value of the benefit supplier recommendation consumption motivation is the largest. We perform *t*-tests on the benefit supplier recommendation consumption motivation with benefit group recommendation consumption motivation, benefit morality recommendation consumption motivation, and benefit demander recommendation consumption motivation, respectively. The results found that the difference in the mean value between benefit supplier recommendation consumption motivation and the other three types of altruistic recommendation consumption motivations was significant (*p* < 0.05), indicating that benefit supplier motivation has the greatest influence among the four kinds of altruistic consumption motivations that affect altruistic recommendation consumption behavior. Therefore, hypothesis H2_d_ holds.

**Figure 1:**
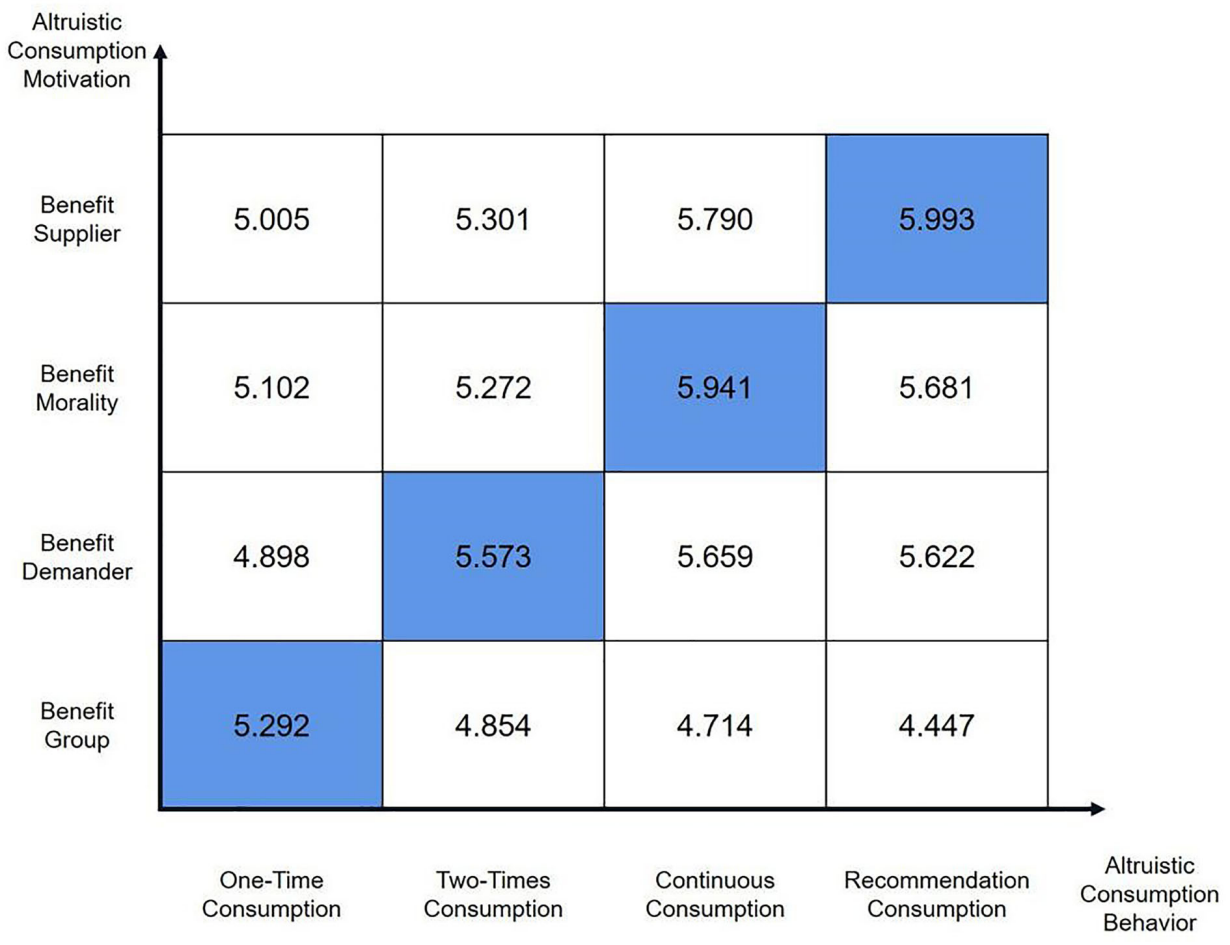
Data matrix of altruistic consumption motivation and altruistic consumption behavior.

In order to show the above data more intuitively, we summarized and plotted the data matrix of altruistic consumption motivation and altruistic consumption behavior (shown in Figure 1).

#### The evolutionary trend of altruistic consumption motivations' influence on altruistic consumption behavior

We performed the dominance analysis and Shapley value decomposition of the four kinds of altruistic consumption motivations' influence on times of altruistic consumption and altruistic consumption behavior. The data results are shown in Tables 5, 6.

**Table 5:** Dominance analysis.

	**Factors**	**Dominance statistics**	**Standardized dominance statistics**	**Ranking**
Times of altruistic consumption	Benefit group motivation	0.028	0.246	3
	Benefit morality motivation	0.029	0.254	2
	Benefit demander motivation	0.041	0.363	1
	Benefit supplier motivation	0.015	0.137	4
Altruistic consumption behavior	Benefit group motivation	0.025	0.357	2
	Benefit morality motivation	0.005	0.073	4
	Benefit demander motivation	0.008	0.111	3
	Benefit supplier motivation	0.032	0.459	1

**Table 6:** Shapley value decomposition.

	**Factors**	**First round effects**	**Shapley value**	**%**
Times of altruistic consumption	Benefit group motivation	25.313	28.255	24.59
	Benefit morality motivation	56.422	29.237	25.44
	Benefit demander motivation	65.869	41.687	36.27
	Benefit supplier motivation	42.737	15.745	13.70
Altruistic consumption behavior	Benefit group motivation	11.660	12.367	35.67
	Benefit morality motivation	3.543	2.525	7.280
	Benefit demander motivation	7.105	3.853	11.12
	Benefit supplier motivation	20.473	15.921	45.93

Among the four kinds of altruistic consumption motivations that affect the times of altruistic consumption, the influence of benefit demander motivation is relatively larger. Dominance analysis of the times of consumers' altruistic consumption and the four kinds of altruistic consumption motivations (shown in Table 5) shows that the benefit demander motivation affects the times of consumers' altruistic consumption most, followed by benefit morality motivation and benefit group motivation, and benefit supplier motivation. That is, among the four altruistic consumption motivations that affect the times of altruistic consumption, the benefit demander motivation has relatively larger influence. In order to get a more intuitive view of the importance of the four altruistic consumption motivations' influence on the times of altruistic consumption, we used Shapley value decomposition and found that the contribution rate of benefit demander motivation to the times of altruistic consumption was 36.270%, the contribution rate of benefit morality motivation to the times of altruistic consumption was 25.440%, the contribution rate of benefit group motivation to the times of altruistic consumption was 24.590%, and the contribution rate of benefit supplier motivation to the times of altruistic consumption was 13.700%. Both test methods show that among the four kinds of altruistic consumption motivations that affect the times of altruistic consumption, benefit demander motivation has a relatively larger influence. Therefore, hypothesis H3_a_ holds.

In the process of statistical analysis, we found that among the four altruistic consumption motivations that affect altruistic consumption behavior, benefit supplier motivation has a relatively larger influence. Dominance analysis of the consumers' altruistic consumption behavior and the four kinds of altruistic consumption motivations (shown in Table 5) shows that the benefit supplier motivation affects the altruistic consumption behavior most, followed by benefit group motivation and benefit demander motivation, and benefit morality motivation. In order to get a more intuitive view of the importance of the four altruistic consumption motivations' influence on the consumers' altruistic consumption behavior, we used Shapley value decomposition and found that the contribution rate of benefit supplier motivation to the altruistic consumption behavior was 45.930%, the contribution rate of benefit group motivation to the altruistic consumption behavior was 35.670%, the contribution rate of benefit demander motivation to the altruistic consumption behavior was 11.120%, and the contribution rate of benefit morality motivation to the altruistic consumption behavior was 7.280%. Both test methods show that among the four kinds of altruistic consumption motivations that affect the consumers' altruistic consumption behavior, benefit supplier motivation has a relatively larger influence.

**Figure 2:**
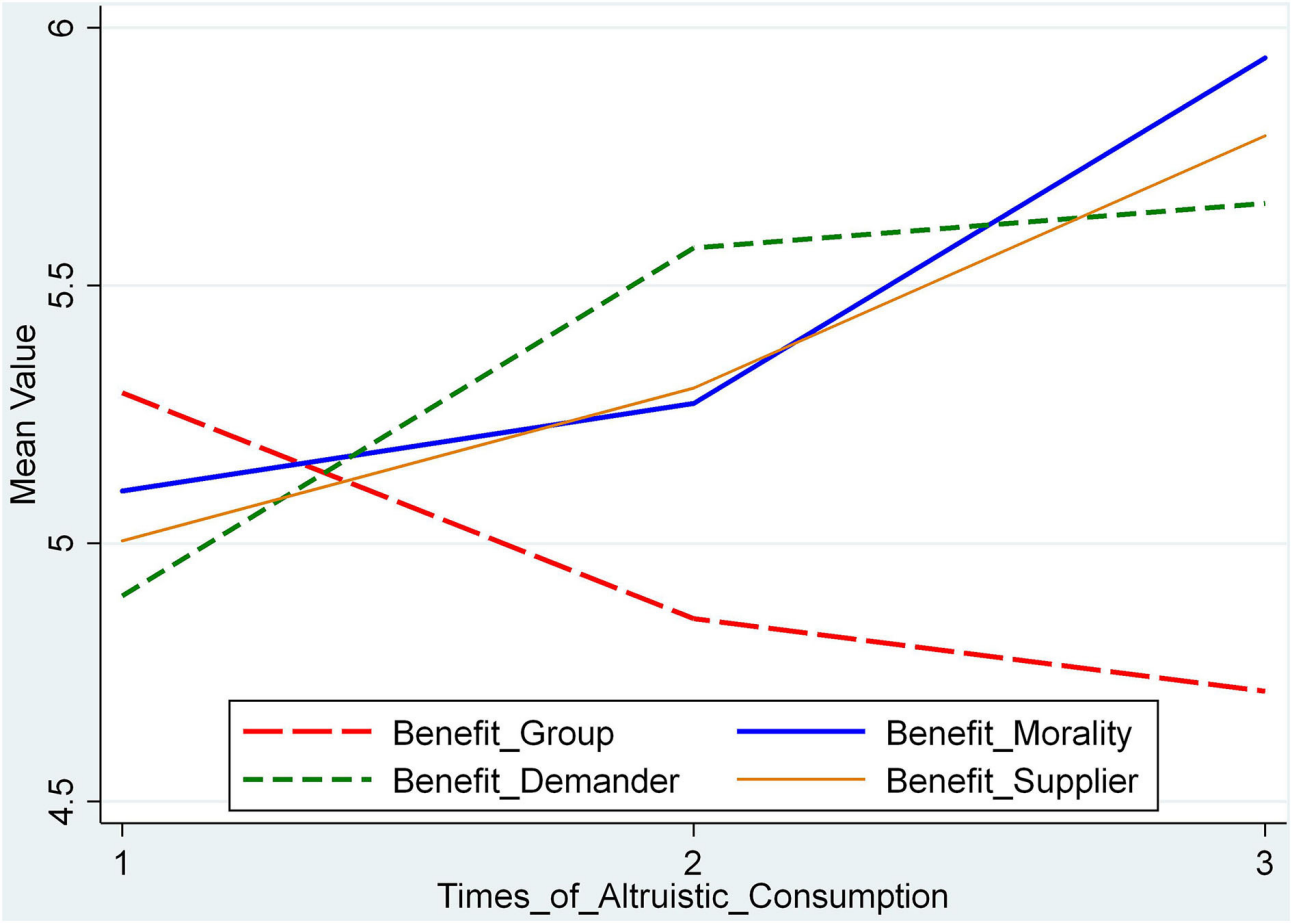
Line chart of altruistic consumption motivation and the times of altruistic purchases.

The strength of benefit group motivation decreases with the increase in the times of altruistic consumption, while the strength of benefit morality motivation, benefit demander motivation, and benefit supplier motivation increases with the increase in the times of altruistic consumption. We take the times of the altruistic consumers' altruistic consumption as the abscissa, and the mean value of the questionnaire items related to the four kinds of altruistic consumption motivations as the ordinate, and made a line chart of the four kinds of altruistic consumption motivations (shown in Figure 2). Therefore, hypothesis H3_b_ holds.

## Discussion

### Research conclusion

This research shows that there is an influential relationship between altruistic consumption motivations and altruistic consumption behaviors that aim for vulnerable groups. Altruistic consumption behavior is mainly affected by benefit group motivation, benefit morality motivation, benefit demander motivation, and benefit supplier motivation. There is a correlation between the four kinds of altruistic consumption behaviors and the four kinds of altruistic consumption motivations. There is a correlation between the four kinds of altruistic consumption behaviors and the four kinds of altruistic consumption motivations. However, in the one-time altruistic consumption behavior, the benefit group motivation has larger influence than the other three motivations, in the two-times altruistic consumption behavior, the benefit demander motivation has larger influence than the other three motivations, in the continuous altruistic consumption behavior, the benefit morality motivation has larger influence than the other three motivations, and in the altruistic recommendation consumption behavior, the benefit supplier motivation has larger influence than the other three motivations. The strength of altruistic consumption motivations changes with the times of altruistic consumption behavior. Among them, the strength of benefit group motivation decreases with the increase of the times of altruistic consumption behavior, while the strength of benefit morality motivation, benefit demander motivation, and benefit supplier motivation increases with the increase of the times of altruistic consumption behaviors. Among the four kinds of altruistic consumption motivations that affect the times of altruistic purchase, the contribution of the benefit demander motivation is relatively larger, and among the four kinds of altruistic consumption motivations that affect the altruistic consumption behavior, the contribution of benefit supplier motivation is relatively larger.

### Practical inspiration

Charitable behavior is a unilateral effort of donors to improve the plight of recipients, while recipients are passive or even inaction recipients, while altruistic consumption fully mobilizes the enthusiasm of beneficiaries of altruistic consumption. Altruistic consumption can lead vulnerable groups into the social reproduction cycle by consuming their yield. Through altruistic beneficiaries' planned, organized, and targeted reciprocal behavior, the sustainability of altruistic consumption behavior can be realized. This research found that consumers' altruistic consumption is based on four kinds of altruistic consumption motivations, so the supplier of altruistic products can adopt different marketing strategies according to the altruistic consumption motivations of different altruistic consumers, changing passive acceptance of charity into active altruistic consumption marketing. Vulnerable groups can use outside support to benefit from their own yield to completely get rid of poverty, replace charity with consumption, and turn themselves from a social burden to a creator of social wealth.

As a new altruistic consumption model, the active marketing of vulnerable groups as the supplier must consider the marketing strategy based on the relationship between altruistic consumption motivation and altruistic consumption behavior.

It is found that the times of altruistic consumption vary from person to person, and it is of great significance for suppliers to study the altruistic consumption motivations of consumers with different consumption times. For one-time altruistic consumption consumers, group promotion is effective. Therefore, suppliers of altruistic consumption products should make full use of their status as vulnerable groups, and make use of the influence of the group initiatives to promote the attempt consumption of their products, promote their products to enter the market, and solve the market entry case of products of vulnerable groups. Our research found that the altruistic consumption behavior dominated by benefit group motivation has a one-time consumption tendency, while the two-times altruistic consumption consumers' benefit demander motivation is relatively stronger, that is to say, the benefit demander motivation is the main factor affecting two-times altruistic consumption behavior. Therefore, in order to make consumers consume again, the supplier should focus on the consumer's demand for the product itself. Only by satisfying the consumer's benefit demander motivation, can the consumer's two-times altruistic consumption of the product be realized, so as to solve the problems of market consolidation for the products of vulnerable groups. Our research found that only meet the demand, the consumption is also difficult to make the continuous altruistic consumption, because there are many products that can meet consumer needs in society and are available everywhere, therefore, in order for consumers to continuous altruistic consumption, in addition to satisfying consumers' benefit demander motivation, suppliers must also meet consumers' benefit morality motivation. Only by letting consumers realize that while consuming the products they need, they can also promote social virtues and fulfill their social responsibilities, will they have the motivation to continuous altruistic consumption, realize the continuous consumption of altruistic products. Only by solving the problem of continuous sales of products of vulnerable groups can the problem of continuous poverty of vulnerable groups be solved, and the return to poverty caused by the consumption relationship dissolution can be prevented, and the true goal of altruistic consumption can be realized. The research also found that among the four kinds of motivations that affect consumers' altruistic recommendation consumption behavior, the benefit supplier motivation is relatively stronger, which means that it is of great significance for more consumers to realize that their consumption behavior can help the vulnerable groups get rid of the vulnerable status, they are willing to recommend to others, and drive more people to consume the altruistic products of the vulnerable groups. Therefore, as a supplier of vulnerable groups, it is necessary to feedback to the society the huge changes brought about by altruistic consumption to vulnerable groups through various channels, so that altruistic consumers know that their altruistic recommendation consumption behavior has produced a positive altruistic effect, which is helpful to encourage altruistic consumers' recommendation consumption behavior. The altruistic marketing strategy adopted for consumers' benefit group altruistic consumption motivation can be called the *benefit group altruistic marketing strategy*, which mainly solves the problem of attempting consumption by altruistic consumers and the problem of entering the market of altruistic products; The altruistic marketing strategy adopted for consumers' benefit demander altruistic consumption motivation can be called the *benefit demander altruistic marketing strategy*, which mainly solves the problem of re-consumption of altruistic products and the problem of market consolidation of altruistic products; The altruistic marketing strategy adopted for consumers' benefit morality altruistic consumption motivation can be called the *benefit morality altruistic marketing strategy*, which mainly solves the problem of continuous consumption and the continuous sales of altruistic products; The altruistic marketing strategy adopted for altruistic recommendation consumption behavior can be called the *benefit supplier altruistic marketing strategy*, which mainly solves the problem of the recommendation consumption of altruistic consumers and the market expansion of altruistic products. We can also collectively refer to these altruistic marketing strategies as “4B altruistic marketing strategies”.

The corresponding relationship between “4B altruistic marketing strategy” and “4B altruistic consumption motivation” is relative. Each marketing strategy can play a role in every altruistic consumption behavior, but the degree of influence is different in different consumption behaviors.

**Figure 3:**
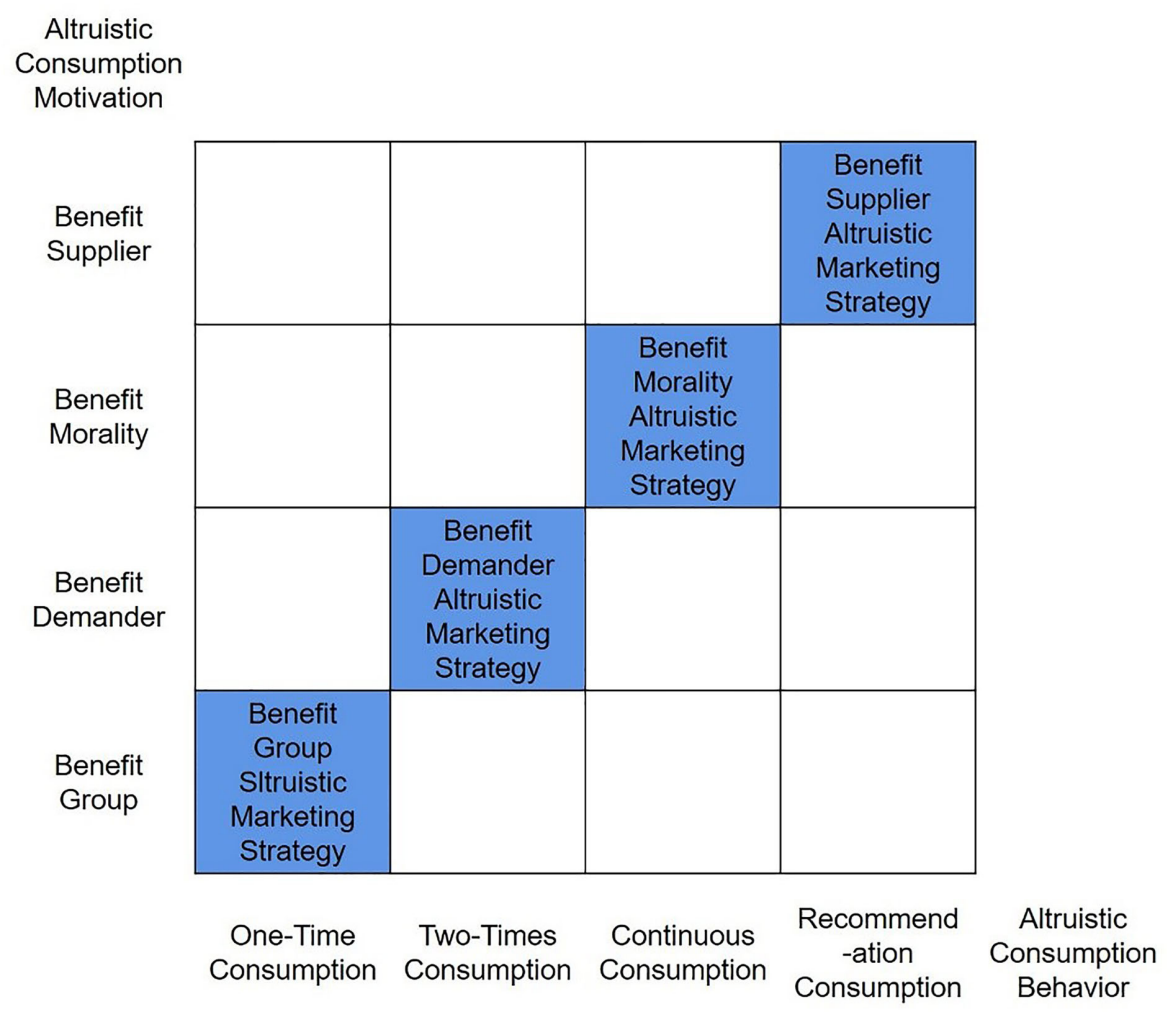
Matrix of altruistic consumption behavior/altruistic consumption motivation and altruistic consumption marketing strategy.

This research summarizes and plotted the matrix of altruistic consumption behavior/altruistic consumption motivation and altruistic consumption marketing strategy (shown in Figure 3).

Benefit group altruistic marketing strategy has the greatest influence on the initial consumption, and as the times of consumption increase, its influence gradually decreases. However, the influence of the benefit demander altruistic marketing strategy, the benefit supplier altruism marketing strategy, and the benefit morality altruism marketing strategy are smaller on the initial consumption, as the times of consumption increases, the influence gradually increases. Therefore, in order to achieve good altruistic marketing effects, suppliers must be good at the combined application of altruistic marketing strategies.

Active marketing of vulnerable groups should also arrange targeted altruistic marketing strategies based on the contribution of altruistic motivations. The research also found that among the four kinds of altruistic consumption motivations that affect consumers' altruistic consumption behavior, the contribution of the benefit supplier motivation is relatively stronger, indicating that the core of altruistic consumption behavior is altruism. Therefore, when formulating altruistic marketing strategies, suppliers must always emphasize the value that altruistic consumption can bring to vulnerable groups, meaning that emphasizes the altruistic nature of consumption, which makes sense for the vulnerable groups to promote the altruistic consumption of their products. However, among the four kinds of altruistic consumption motivations that affect the times of consumers' altruistic consumption, the contribution of benefit demander consumption is relatively stronger. This tells us that altruistic consumption is not a kind of charitable behavior, but a reciprocal altruistic behavior. Therefore, the product must meet the consumer's own needs, that is, satisfy the consumer's benefit demander motivation. Therefore, improving the quality of altruistic consumption products, improving consumer services, setting appropriate prices, and providing convenient channels are essential to prevent the consumption relationship dissolution, maintain the stability of the consumer market, and promote the continuous consumption of altruistic consumption products.

## Limitations and prospects

This research focuses on the relationship between altruistic consumption motivation and altruistic consumption behavior. Regarding the altruistic consumption model, the research team has completed relevant research and published articles, so there is not much involved in this research. This research only studied the motivations of different individuals' one-time, two-times, and continuous consumption, while did not study the motivations of the same individual's first, second, and continuous consumption. Therefore, this research cannot answer the question of why an individual decides to consume again after the first consumption, and why an individual decides to consume continuously. Researching these motivations is also of great significance for expanding the altruistic consumer market. This research is investigative research, and the object of research is consumption motivation. Consumers' motivations are inherent and cannot be directly expressed through consumers' consumption behavior, nor can they be derived from actual statistics data. This study focuses on consumers who buy poverty alleviation products and on vulnerable groups with productive capacity in China. Whether the study represents all altruistic consumers and vulnerable groups requires further investigation.

The development of real society is always uneven, there are always a large number of groups that in the countries, regions, ethnic minorities, low-income groups, and affected by disasters that in a state of poverty and backwardness. In the long run, it is challenging to help vulnerable groups out of poverty using the one-way charity model. Adopting the reciprocal altruistic consumption method can be an effective way to help vulnerable groups escape poverty. Using marketing methods to help vulnerable groups is an essential extension of the theory of cause-related marketing, a new field of marketing study, and a manifestation of the new function of marketing.

## Data availability statement

The raw data supporting the conclusions of this article will be made available by the authors, without undue reservation.

## Ethics statement

Ethical review and approval was not required for the study on human participants in accordance with the local legislations and institutional requirements. Written informed consent to participate in this study was provided by the participants' legal guardian/next of kin.

## Author contributions

HX and CL designed and conducted the study. All authors contributed to the article and approved the final version.

## Conflict of interest

The authors declare that the research was conducted in the absence of any commercial or financial relationships that could be construed as a potential conflict of interest.

## Publisher's note

All claims expressed in this article are solely those of the authors and do not necessarily represent those of their affiliated organizations, or those of the publisher, the editors and the reviewers. Any product that may be evaluated in this article, or claim that may be made by its manufacturer, is not guaranteed or endorsed by the publisher.
